# Marijuana-Derived Cannabinoids Trigger a CB2/PI3K Axis of Suppression of the Innate Response to Oral Pathogens

**DOI:** 10.3389/fimmu.2019.02288

**Published:** 2019-10-15

**Authors:** Zhen Gu, Shilpa Singh, Rajarshi G. Niyogi, Gwyneth J. Lamont, Huizhi Wang, Richard J. Lamont, David A. Scott

**Affiliations:** Oral Immunology and Infectious Diseases, University of Louisville School of Dentistry, Louisville, KY, United States

**Keywords:** cannabinoid receptor type 1 and 2 (CB1, CB2), cholinergic anti-inflammatory system, glycogen synthase kinase (GSK3β), inflammation, oral bacteria, phosphatidylinositol 3-phosphate-kinase (PI3K), periodontitis, phytocannabinoid

## Abstract

Cannabis use is an emergent risk factor for periodontitis, a chronic bacterial-induced disease of the supporting structures of the teeth. However, the mechanisms by which marijuana exposure predisposes to periodontal tissue destruction have yet to be elucidated. Therefore, we examined the influence of physiologically relevant doses of major marijuana-derived phytocannabinoid subtypes (cannabidiol [CBD]; cannabinol [CBN]; and tetrahydrocannabinol [THC], 1.0 μg/ml) on the interactions of three ultrastructurally variant oral pathogens, *Porphyromonas gingivalis, Filifactor alocis*, and *Treponema denticola* with the immune system. CBD, CBN, and THC each suppressed *P. gingivalis*-induced IL-12 p40, IL-6, IL-8, and TNF release while enhancing the anti-inflammatory cytokine, IL-10, from human innate cells. Similar phenomena were observed in *F. alocis-* and *T. denticola*-exposed human monocytes and human gingival keratinocytes. Higher phytocannabinoid doses (≥5.0 μg/ml) compromised innate cell viability and inhibited the growth of *P. gingivalis* and *F. alocis*, relative to unexposed bacteria. *T. denticola*, however, was resistant to all cannabinoid doses tested (up to 10.0 μg/ml). Pharmaceutical inhibition and efficient gene silencing indicated that a common CB2/PI3K axis of immune suppression is triggered by phytocannabinoids *in vitro*. This pathway does not appear to perpetuate through the canonical GSK3β-dependent cholinergic anti-inflammatory pathway, the predominant endogenous inflammatory control system. In a repetitive, transient oral infection model, CBD also suppressed *P. gingivalis*-induced innate immune markers in wild-type mice, but not in CB2^−/−^ mice. If such phenomena occur in humans *in situ*, environmental cannabinoids may enhance periodontitis via direct toxic effects on specific oral bacteria; by compromising innate cell vitality; and/or through a suppressed innate response to periodontal pathogens involving a CB2/PI3K signaling lineage.

## Introduction

Approximately 12% of the N. American and 4% of the global adult population are cannabis users (1). With the number of legislative bodies decriminalizing medicinal and recreational possession likely to grow, the percentage of marijuana users could increase concomitantly worldwide. Further, phytocannabinoid oils, particularly cannabidiol (CBD), are increasing in popularity. However, despite centuries of use, the influences of marijuana and phytocannabinoids on the immune system remain unclarified.

Periodontitis is a chronic, highly prevalent destructive, bacteria-initiated, chronic inflammatory disease of the soft and hard tissues surrounding the teeth. Periodontitis-associated spending consumes a considerable share of an annual oral disease economic burden of >US$400B (2), while > 500M people are estimated to be effected by severe periodontitis (3). While the role of the endocannabinoid system on gingival health is not yet well-understood (4), recent studies have consistently suggested that marijuana is an important risk factor for periodontitis in humans (5–15). Tobacco-independent relative risk for marijuana use has been estimated at between 1.6 and 3.1, depending on the disease severity threshold assessed, which is similar to tobacco smoke itself (5, 8, 12). The tobacco-cannabis combined risk has been estimated to be 2.5 [1.5–4.5] (12). Importantly, destructive periodontal diseases in marijuana users may onset at an earlier age than seen in the general periodontitis population, who do not normally develop periodontitis until middle age (6–8, 11–13). While Osola et al. have suggested that the CB2 agonist, HU-308 is osteoprotective in an LPS-induced oral inflammation model in rats (16), other studies suggest that cannabinoids may similarly predispose animals to destructive periodontal disease. For example, rodents with ligated teeth exposed to marijuana smoke for only 8 min per day develop more extensive alveolar bone loss than ligated, unexposed animals (17). Reduced bone healing around implants has also been demonstrated in cannabis smoke-exposed animals (18). The mechanisms underlying predisposition to destructive periodontal disease due to marijuana exposure are unclear, although cannabis has long been ascribed anti-inflammatory properties.

Innate immune cells express the cannabinoid receptor, CB2, while exogenous cannabinoids have been reported to compromise host resistance to specific infectious agents, including *Listeria monocytogenes* (19, 20). Therefore, we set out to determine if, and how, physiologically relevant concentrations (21) of representatives of the three major marijuana-derived phytocannabinoid subtypes (CBD; cannabinol [CBN]; and tetrahydrocannabinol [THC]) may suppress the inflammatory response of human monocytes and oral epithelial cells to, and growth characteristics of, three representative oral pathogens, *Porphyromonas gingivalis, Filifactor alocis*, and *Treponema denticola*. General anti-inflammatory properties of CBD were also assessed in control and CB2^−/−^ mice orally infected with *P. gingivalis*. *P. gingivalis* is the archetypal Gram-negative, anaerobic periodontal pathogen that has also been associated with several systemic sequelae of periodontitis, including poor pregnancy outcome, cardiovascular complications, Alzheimer's disease and, as we have recently shown, esophageal cancer (22–26). *T. denticola*, a highly motile anaerobic spirochete, is a long established periodontal pathogen that can be a major component of the subgingival biofilm (26, 27). *F. alocis*, a Gram-positive anaerobe, has recently emerged from several microbiome studies as a potentially key periodontal pathobiont (28, 29).

Mammals have developed multiple endogenous mechanisms to promote innate response homeostasis by containing the inflammatory response to encounters with pathogens. These include both direct innate suppression pathways, most notably the acetylcholine- (and nicotine/cotinine-) activated cholinergic anti-inflammatory axis (30–32), and inflammation resolution pathways, triggered by mediators such as resolvins and maresins (33–35). A central regulator common to many of such innate dampening mechanisms is the constitutively active, phosphorylatively inactivated inflammatory gatekeeper, GSK3β (34, 36). GSK3β targeting leads to suppression of TLR-initiated pro-inflammatory cytokines under NF-κB p65 transcriptional control and augmentation of IL-10 via promotion of CREB-dependent gene activation (31, 36), sufficient to protect from LPS-induced septic shock (31). In mice, GSK3β inhibition suppresses the local and systemic inflammatory response to oral infection with *P. gingivalis*, as we have previously shown (37). Cannabinoids have also been noted to suppress neural inflammation in a GSK3β-related manner (38). Thus, we also set out to examine the immunosuppressive influence of cannabinoids in the context of the TLR-PI3K-GSK3β signaling axis using a combinatorial pharmaceutical inhibition and gene silencing strategy. We report that, while cannabinoids are potent pro-inflammatory suppressors and IL-10 inducers, their mode of action involves a CB2/PI3K signaling lineage that does not amplify the canonical GSK3β anti-inflammatory pathway. These data provide mechanistic insight into how marijuana and cannabinoids may be therapeutically exploited to suppress inflammation, when this is a clinically desirable outcome. On the other hand, they afford an increased understanding of how phytocannabinoids exposure may predispose to chronic bacterial-induced diseases, such as periodontitis.

## Methods and Methods

### Materials and Animals

*Porphyromonas gingivalis* ATCC 33277, *Filifactor alocis* ATCC 35896, *Treponema denticola* ATCC 35405 and telomerase-immortalized gingival keratinocytes (TIGK) were revived from laboratory frozen stocks. Gifu anaerobic broth (GAM) and brain heart infusion (BHI) medium were purchased from Nissui Pharmaceutical (Tokyo, Japan) and Becton Dickinson (Sparks, MD), respectively. Ultrapure LPS from *Escherichia coli* 0111:B, trypan blue, the serine and cysteine protease/gingipain inhibitor tosyl-L-lysine chloromethylketone, gelatin, volatile fatty acids (glacial acetic, propionic, N-butyric, N-valeric, isobutyric, isovaleric, and DL-methylbutyric acids), carboxymethyl cellulose salt, methanol, isoflurane, RNAlater, agarose, methylene blue, eosin, hematoxylin, L-cysteine, and arginine were bought from Sigma-Aldrich (St. Louis, MO). Monocyte isolation kits came from Miltenyi Biotec (Auburn, CA). Rabbit serum and tryptone was purchased from ThermoFisher (Waltham, MA). Tryptone came from Fisher Scientific (Fair Lawn, NJ). DermaLife keratinocyte medium was from Lifeline Cell Technology (Walkersville, MD). RPMI Complete was purchased from Invitrogen Life Technologies (Carlsbad, CA). Murine PI3K p85α-, PI3K p110δ-, and β-actin-specific antibodies, lupine anti-GAPDH, caprine anti-mouse IgG-HRP and anti-rabbit IgG-HRP antibodies were from Cell Signaling Technology (Danvers, MA, USA). Rabbit polyclonal anti-cannabinoid receptor 1 (CB1) and anti-cannabinoid receptor 2 (CB2) antibodies came from Abcam (Cambridge, MA, USA). Cannabidiol (CBD), cannabinol (CBN), and Δ^9^-tetrahydrocannabinol (THC) were purchased from Cayman Chemical Co. (Ann Arbor, MI, USA). Enhanced chemiluminescence kits came from Thermo Scientific (Rockford, IL). Cytokine ELISA kits (IL-6, IL-8, IL-10, TNF [TNF-α]) were purchased from eBioscience (San Diego, CA) while IL-12 p40 ELISA kits came from Boster Immunoleader (Pleasanton, CA, USA). The highly selective cannabinoid CB2 inverse agonist, JTE907, the GSK3β inhibitor, SB216763, and the PI3K inhibitor, LY294002, came from Tocris Biosciences (Minneapolis, MN, USA). Non-targeted Signal Silence Control siRNA, CB1 siRNA, CB2 siRNA, PIK3R1 (p85α) siRNA, and PIK3CD (p110δ) siRNA were purchased from Dharmacon (Lafayette, CO, USA). LipoJet™ *in vitro* siRNA transfection kit (Ver.2) came from SignaGen Laboratories (Rockville, MD, USA). P3 primary cell 4D-Nucleofector X kits were from Lonza BioResearch (Allendale, NJ, USA). 3-(4,5-dimethylthiazol-2-yl)-2,5-diphenyltetrazolium bromide (MTT) cell viability assay kit was from Molecular Probes (Waltham, MA, USA). Wizard® Genomic DNA Purification kits were from Promega (Madison, WI). C57BL6 wild type and CB2 receptor deficient mice were purchased from the Jackson Laboratory (Bar Harbor, ME). Oral gavage needles were obtained from Cadence Science Inc. Cranston, RI). Buffer RLT and RNeasy kits came from Qiagen (Germantown, MD). CO_2_ was provided by Welders Supply Co. (Louisville, KY).

### Growth of Bacteria

*P. gingivalis, F. alocis*, and *T. denticola* were grown in GAM, BHI supplemented with L-cysteine (0.1%) and arginine (20%) and TYGVS (39), respectively, under anaerobic conditions (80% N_2_, 10% H_2_, 10% CO_2_) at 37°C. Bacteria were harvested at mid to late log phase, as determined spectrophotometrically (O.D. _600nm_). *P. gingivalis, F. alocis*, and *T. denticola* were also grown in the presence or absence of phytocannabinoids (0.0–10.0 μg/ml), including the appropriate solvent controls.

### Isolation of Human Monocytes

Primary human monocytes were purified from anonymized, citrated whole blood by using anti-CD14 microbeads or by depleting non-monocytes, as we have previously reported (31, 40) and as approved by the University of Louisville, Institutional Review Board, #12.0346. This procedure routinely results in >95% pure CD14^+^ cells, as shown by flow cytometry. Human monocytes were cultured at 37°C and 5% CO_2_ atmosphere, in complete RPMI plus or minus stimulating agents, as described below. Monocyte viability was determined by trypan blue exclusion and MTT assays.

### Growth of Human Gingival Epithelial Cells

Human telomerase-immortalized gingival keratinocytes (TIGKs), derived from a primary gingival epithelial cell line, were maintained in supplemented DermaLife keratinocyte medium, as described previously (41). Cells between passages 10 and 20 were cultured to 80% confluence prior to exposure to LPS or oral bacteria (5% CO_2_, 37°C).

### Cytokine Release by Innate Cells

Human monocytes (2 × 10^5^ cells/well) or TIGK cells (2 × 10^4^ cells per well) were exposed to CBD; CBN; or THC (0–10 μg/ml), including the appropriate solvent controls, for 2 h in order to test phytocannabinoid cytotoxity and dose-related suppression of LPS-induced cytokine suppression. TIGK and monocyte viability was compromised at phytocannabinoid concentrations of 10 μg/ml but not 5.0 μg/ml or below. Further, phytocannabinoids effectively suppressed LPS-induced cytokine suppression at 0.1 and 1.0 μg/ml. Therefore, unless otherwise stated, subsequent monocytes and TIGKs exposures were performed at CBD; CBN; or THC concentrations of 1.0 μg/ml prior to stimulation, or not, with LPS (0.1 μg/ml); *P. gingivalis* (MOI, 1–50:1); *F. alocis* MOI, 1–50:1); or *T. denticola* (MOI, 1–50:1) in the context of CB2 (JTE 907, 0–10 μg/ml), P13K (LY294002, 0–10 μM) or GSK3β (SB216763, 0–10 μM and LiCl, 0–10 mM) inhibition. Solvent controls were employed, as appropriate, throughout. Cell-free supernatants were harvested at 20 h and assayed for cytokine levels by ELISA. TLCK (50 μM) was employed to minimize proteolytic degradation of secreted cytokines.

### Silencing of Genes Encoding CB1, CB2, PI3K p110δ/p85α, and GSK3β

Transfection of CB1 and CB2 siRNA to human monocytes or TIGK cells was carried out using the LipoJet™ *in vitro* siRNA transfection kit while electroporation transfection of PI3K p110δ/p85α and GSK3β siRNA to human monocytes was carried out using Lonza Nucleofector technology (Allendale, NJ, USA), according to the manufacturer's protocols. The levels of total CB1, CB2, PI3K p110δ/p85α, and GSK3β were assessed by Western blot at 48 h post-transfection. Cells were subsequently exposed to CBD (1.0 μg/ml) for 2 h, then LPS (0.1 μg/ml) for 20 h and the supernatants were harvested for cytokine assay by ELISA.

### CBD-Innate Interactions in a Murine Oral Gavage Infection Model

To assess the influence of CBD on the inflammatory response to oral bacterial infection, an adopted Baker model (42) with repeated transient *P. gingivalis* infections was employed. After a 5 day acclimatization period, male, randomized CB2^−/−^ and subsequently age-matched wild type C57Bl6 mice were administered cannabidiol (10 mg/kg) or vehicle only every other day until sacrifice at Day 48. CBD dosage was based on prior studies that employed this specific cannabinoid, also i.p., to suppress microbial insult-induced inflammatory regulators in murine models of neuronal and pulmonary disease (43, 44). As C57Bl6 are not a natural *P. gingivalis* host, the mice were repeatedly infected with *P. gingivalis* in 2% carboxymethyl cellulose in PBS (CMC; 10^9^ cfu/ml, 100 μl inocula), or administered CMC only, via a 22G oral gavage needle on Day 6 and alternate days for five infection cycles. Thus, there were four groups of five mice each. Mice were euthanized (CO_2_ at 1.5 l/min; followed by cervical dislocation) 42 days after the final *P. gingivalis* infection. *P. gingivalis* infection was monitored by culture on blood agar plates and by PCR from maxillary swabs collected 8 days after first infection and then every 2 weeks after last infection. All experimental procedures were performed in accordance with the Guidelines of the Institutional Animal Care and Use Committee of University of Louisville (IACUC #15434). Total and *P. gingivalis*-reactive IgG and IgM production was measured in serum by ELISA. Gingiva of the entire maxilla were excised, immersed in buffer RLT and stored at −80°C until RNA isolation using RNeasy, according to the manufacturer's protocol. Gingival inflammatory markers (CD14, CD45, IL-1β, MIP-2, and MMP-8) were subsequently assessed by qPCR. Alveolar bone loss was measured in defleshed, 3% bleach-treated skulls stained with stained with 0.5% eosin (5 min) followed by 1% methylene blue (1 min) using a SMZ 800 dissecting microscope (Nikon Instruments Inc., Melville, NY) fitted with a VIA-170K video image marker measurement system (Boeckeler Instruments Inc., Tucson, AZ). Bone loss was measured at 14 predetermined points on the maxillary molars of de-fleshed maxillae, determined as the distance from the cementoenamel junction to the alveolar bone crest. Potential volumetric and densitometric alveolar bone changes were also assessed using a Skyscan1174 x-ray microtomograph and associated software (Bruker Corporation, Billerica, MA).

### Statistical Analyses

Unless otherwise stated, statistical significance between groups was evaluated by analysis of variance and the Tukey multiple comparison test using the Instat v3.10 program (GraphPad, San Diego, CA). Differences between groups were considered significant at the level of *p* < 0.05.

## Results

### High Doses of Phytocannabinoids Suppress the Growth of *P. gingivalis* and *F. alocis* but Not *T. denticola*

**Figure 1:**
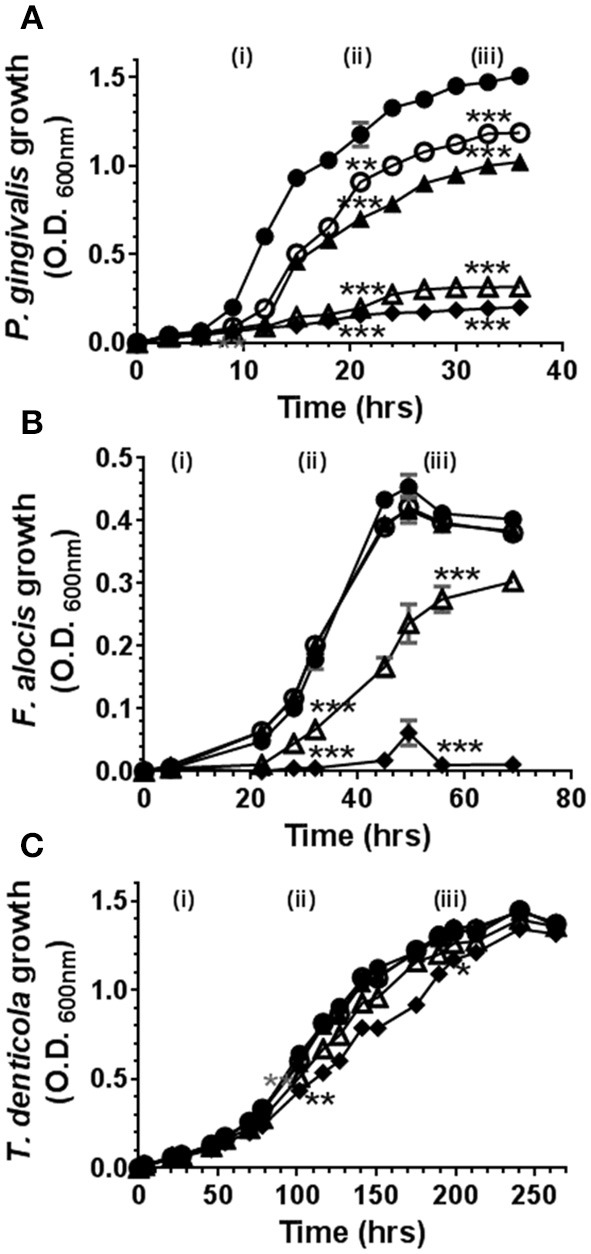
High doses of cannabidiol suppress the growth of **(A)** *P. gingivalis* and **(B)** *F. alocis* but not **(C)** *T. denticola*. *P*. *gingivalis, F*. *alocis*, and *T*. *denticola* were each cultured under anaerobic conditions in the presence or absence of CBD, at the doses noted below, and growth monitored by densitometry. CBN and THC exhibited similar anti-bacterial activities (*data not shown*). Data represent the mean ± SEM of three biological replicates. ●, ○, ▲, Δ, and ♦ represent 0.0, 0.1, 1.0, 5.0, and 10.0 μg/ml CBD, respectively. */**/***p ≤ 0.5/0.01/0.001 respectively, as determined by ANOVA at early-, mid-, and late-log growth phases.

Cannabinoids have, historically, been ascribed antimicrobial properties (45–47). Therefore, we firstly examined the influence of marijuana-derived cannabinoid-subtypes on three important oral pathogens. As shown in Figure 1, high doses of CBD, CBN, and THC (5–10 μg/ml) suppressed the growth of *P. gingivalis* and *F. alocis*. However, *T. denticola* was resistant to each phytocannabinoid at all doses tested (0.1–10 μg/ml). The same general anti-bacterial profile was noted for both CBN and THC (*data not shown*). Thus, three major cannabinoids each display selective anti-microbial activity against key components of the subgingival microbiota.

### High Doses of Phytocannabinoids Reduce Innate Cell Viability

**Figure 2:**
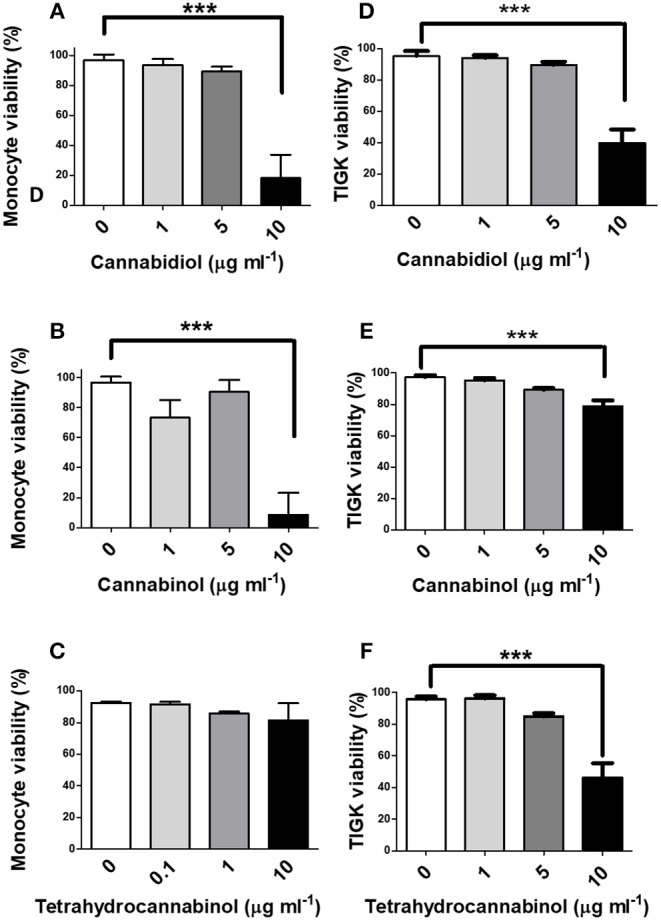
High doses of phytocannabinoids reduce innate cell viability. Primary human monocytes were cultured in the absence or presence of varying concentrations (0–10 μg/ml) of **(A)** CBD, **(B)** CBN, or **(C)** THC and viability established by MTT assay. TIGK cells were also cultured in the absence or presence of varying concentrations (0–10 μg/ml) of **(D)** CBD, **(E)** CBN, or **(F)** THC and viability established by trypan blue exclusion. Data represent the mean ± SD of three biological replicates. ***p ≤ 0.001, as determined by ANOVA.

Next, we investigated the influence of phytocannabinoids on innate cell viability. As shown in Figure 2, high doses of CBD and CBN (10 μg/ml), but not THC, reduced the viability of primary human monocytes. THC has a lower CB2 affinity than either CBD or CBN (48). High doses of CBD, CBN and THC similarly reduced the viability of TIGK cells. Therefore, should these phenomena persist *in situ*, phytocannabinoids have the potential to compromise the viabilty of innate cells that play critical roles in pathogen control, in addition to the prospect of promoting resistant bacterial components of dental plaque.

### Phytocannabinoids Alter Innate Cell Cytokine Release Profile in Response to Oral Bacteria

Cannabis has long been ascribed anti-inflammatory properties (49, 50), although the underlying mechanisms have yet to be convincingly discerned. In initial experiments, we established that CBD, CBN and THC were each cytotoxic to human monocytes at high concentrations (10 μg/ml), as determined by MTT and/or trypan blue exclusion assays, while efficient suppression of LPS-induced cytokine release could be attained at sub-toxic doses of 0.1–1.0 μg/ml. Therefore, all subsequent experiments involving monocytes were performed at a phytocannabinoid concentration of 1.0 μg/ml, unless otherwise stated.

**Figure 3:**
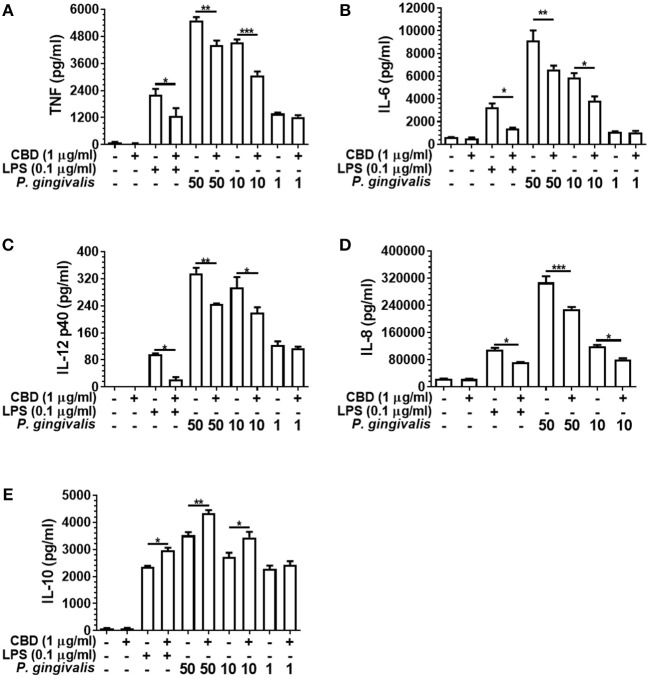
Phytocannabinoids alter the monocytic cytokine release profile in response to oral bacteria. Control and CBD (2 h) pre-exposed monocytes were stimulated, or not, with LPS or *P. gingivalis* and cytokine [**(A)** TNF, **(B)** IL-6, **(C)** IL-12 p40, **(D)** IL-8, **(E)** IL-10] release measured in 20 h cell-free supernatants by ELISA. Similar data was found upon *F. alocis* and *T. denticola* stimulation (*data not shown*). Similar phenomenon were also apparent with two the other phytocannabinoids tested, CBN and THC (*data not shown*). Data represent the mean ± SD of three biological replicates with paired data-points analyzed by *t*-test. */**/***p ≤ 0.05/0.01/0.001.

As presented in Figure 3, CBD suppressed the release of TNF, IL-6, IL-12 p40, and IL-8 from LPS- or *P. gingivalis*-stimulated monocytes, while enhancing IL-10 release. This is consistent with a GSK3β-mediated immune suppression (36). CBD also suppressed the release of the key pro-inflammatory cytokines by *F. alocis*- or *T. denticola*-stimulated monocytes (*data not shown*). Further, similar cytokine profiles were observed for CBN- and THC-exposed monocytes, while CBD, CBN, and THC also suppressed IL-6 release and enhanced IL-10 release from *P. gingivalis*-exposed TIGK cells (*data not shown*). Therefore, different cannabinoid subtypes are each potent suppressors of the innate response to variant bacterial challenges. As is also apparent in Figure 3, there was a clear dose-relationship between the intensity of the inflammatory signal and the ability of cannabinoids to suppress or augment the pro- and anti-inflammatory output, respectively. Further, at the non-cytotoxic doses of phytocannabinoids of 0.1, 1.0, and 5.0 μg/ml, there was a general dose-related suppression of the TNF signal generated in response to a constant inflammatory stimulus of 0.1 μg/ml LPS (*data not shown*). We, next, set out to elucidate the essential signaling events explaining phytocannabinoid-induced innate suppression.

### Inhibition or Silencing of CB2 Abrogates CBD-Mediated Innate Suppression

**Figure 4:**
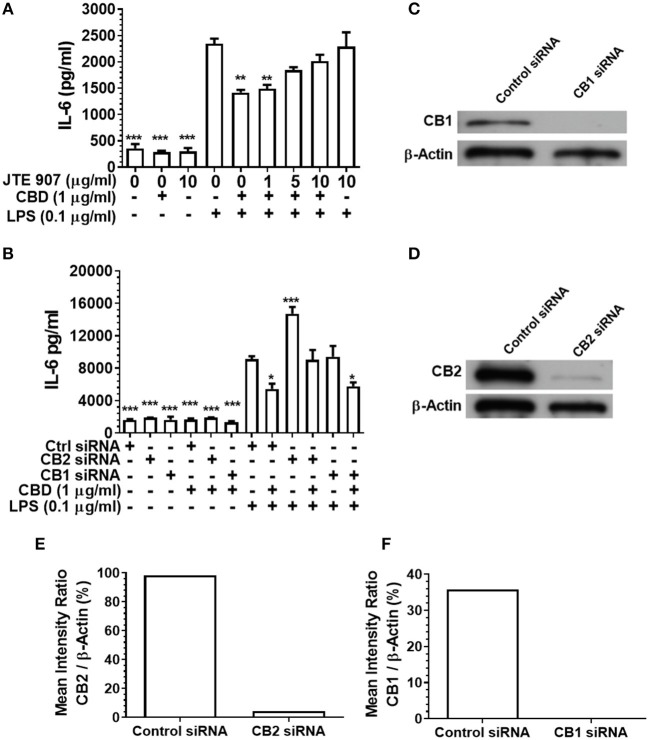
Inhibition or silencing of CB2 abrogates CBD-mediated innate suppression. IL-6 concentrations in 20 h cell-free supernatants were collected from primary human monocytes stimulated, or not, with LPS (0.1 μg/ml) in the presence or absence of CBD **(A)** with and without CB2 blockade by JTE907 or **(B)** with or without CB1 or CB2 silencing using siRNA. Efficient knockdown of **(C,E)** CB1 and **(D,F)** CB2 was confirmed using Image J software. **(A,B)** Data represent the mean ± SD of three biological replicates. **(C–F)** Representative data are presented. */**/***p ≤ 0.05/0.01/0.001, compared to **(A)** LPS-stimulated control or **(B)** control siRNA, LPS-stimulated cells.

Having established that CBD, CBN, and THC each subdue pro-inflammatory cytokine release from monocytes and TIGK cells exposed to oral bacteria, we examined the relative contributions of the cannabinoid receptors CB1 and CB2 to this immunosuppressive phenomenon. Inhibition of the CB2 receptor with JTE907 rescued the innate response of CBD-exposed monocytes to the classic TLR4-agonist, LPS (Figure 4A). Similarly, efficient CB2, but not CB1, gene silencing (Figures 4C–F) rescued the CBD abrogated innate response, as established by IL-6 secretion (Figure 4B). CB2 gene silencing similarly rescued the CBD-inhibited TIGK pro-inflammatory cytokine response to LPS *(data not shown)*. Therefore, monocytic CB2, but not CB1, receptors are critical to the skewed cytokine response to bacterial stimuli noted in cannabinoid-exposed cells.

### Inhibition or Silencing of PI3K Abrogates CBD-Mediated Innate Suppression

**Figure 5:**
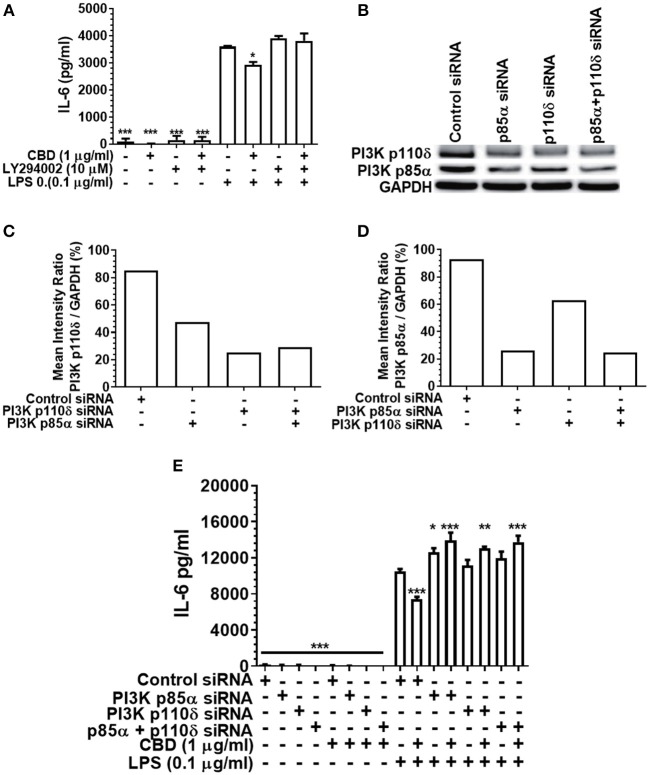
Inhibition or silencing of PI3K abrogates CBD-mediated innate suppression. IL-6 **(A,E)** concentrations in 20 h cell-free supernatants were collected from primary human monocytes stimulated, or not, with LPS (0.1 μg/ml) in the presence or absence of CBD **(A)** with and without CB2 blockade by LY294002; or **(E)** with or without PI3K p110δ and/or p85α silencing using siRNA. Efficient PI3K knockdown was confirmed using Image J software **(B–D)**. Similar data were obtained for IL-8 (*data not shown*). **(A,E)** Data represent the mean ± SD of three biological replicates. **(B–D)** Representative data are presented. */**/***p ≤ 0.05/0.01/0.001, compared to **(A)** LPS-stimulated control or **(E)** control siRNA, LPS-stimulated cells.

As PI3K activation is a critical early event in the cholinergic and other anti-inflammatory signaling cascades (36, 51), and as CBD has been reported to be a PI3K activator in the context of neuronal dysfunction (38), we next set out to elucidate the importance of PI3K in cannabinoid-induced immune suppression. Either pharmacological inhibition or siRNA-mediated silencing of PI3K (Figure 5) rescued the pro-inflammatory response of CBD-exposed, LPS-stimulated monocytes. Thus, cannabinoid-mediated innate suppression is propagated through a CB2-PI3K signaling axis, a step in keeping with established anti-inflammatory signaling chains (31, 36).

### Inhibition of GSK3β Does Not Rescue CBD-Mediated Innate Suppression

**Figure 6:**
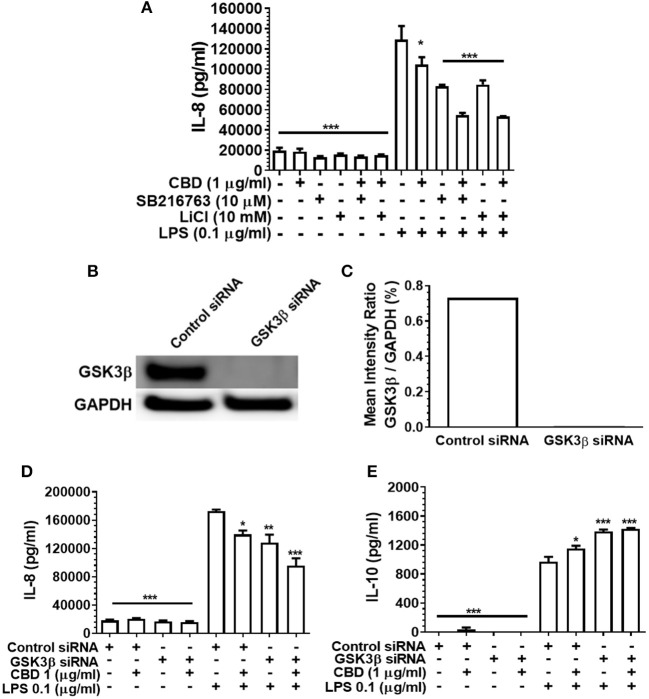
Phytocannabinoid diversion of the innate response to oral bacteria is not GSK3β-dependent. IL-8 concentrations in 20 h cell-free supernatants were collected from primary human monocytes stimulated, or not, with LPS (0.1 μg/ml) in the presence or absence of CBD, with and without GSK3β blockade by SB216763 or by LiCl **(A,D)**. The respective suppressive and enhancing influence of GSK3β silencing on LPS- and/or CBD-exposed monocyte release of IL-8 **(D)** and IL-10 **(E)** are also presented. Efficient GSK3β knockdown of CB2 was confirmed using Image J software **(B,C)**. Data represent the mean ± SD of three biological replicates. */**/*** *p* ≤ 0.5/0.01/0.001, compared to control siRNA, LPS-stimulated cells.

GSK3β is a constitutively active, central mediator of the innate response to TLR agonists and to oral bacteria (36, 51) and cannabinoids have been reported to suppress neural inflammation in a GSK3β-related manner (38). Therefore, we went on to examine the role of GSK3β in phytocannabinoid-induced suppression of inflammation by employing two established GSK3β inhibitors, SB216763 and LiCl. As presented in Figure 6A, neither pharmacological antagonist of GSK3β influenced the efficacy of CBD in repressing the pro-inflammatory cytokine response to LPS. In keeping with the pharmacological data, although efficient GSK3b knock down was apparent (Figures 6B,C), such a gene silencing approach did not influence CBD-mediated innate suppression (Figure 6D) or IL-10 enhancement (Figures 6D,E). Thus, phytocannabinoid-mediated innate response dampening appears to be independent of the central endogenous immune homeostasis controller, GSK3β.

### CBD Suppresses Oral Bacterial-Induced Inflammation *in vivo*

**Figure 7:**
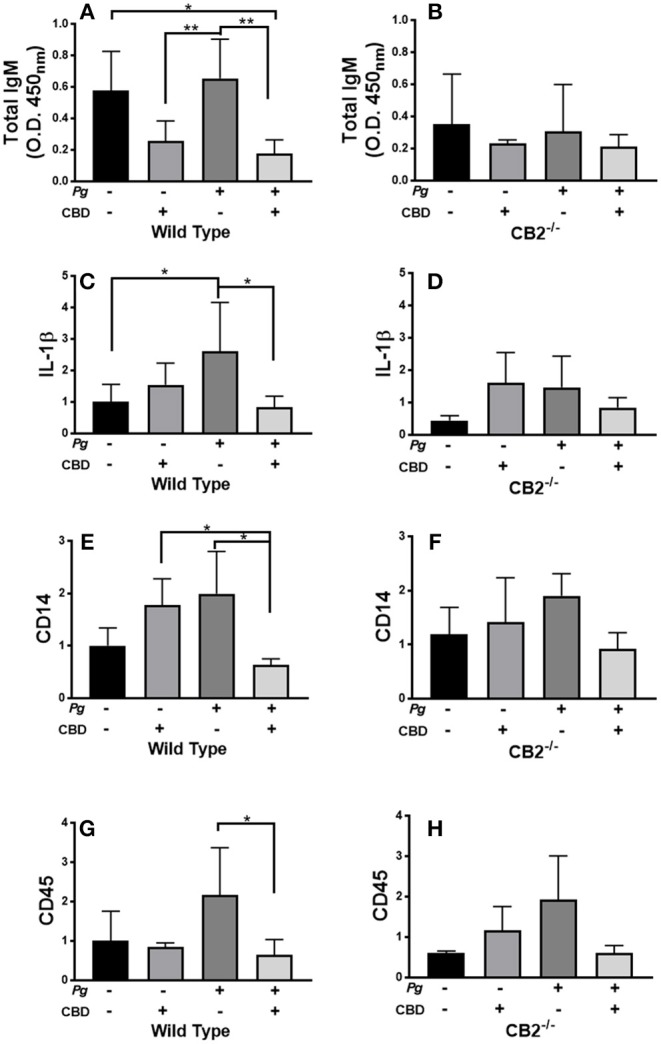
CBD suppresses inflammation induced by repeated oral inoculation with *P. gingivalis*. CB2^−/−^ and control groups of C57Bl6 mice (*n* = 5 per group) were treated with cannabidiol or vehicle control and repeatedly orally infected with *P. gingivalis* 33277 or carboxymethyl cellulose control. Total serum IgM was measured by ELISA **(A,B)**; and gingival tissue transcripts of inflammation-related biomarkers were detected by qPCR **(C–H)**. There were no differences in the total IgG or *P. gingivalis*-cognizant IgM or IgG titers of WT vs. CB2^−/−^ animals (*data not shown*). Total IgM, IL-1β, CD14, and CD45 signals differed in WT mice treated with *P. gingivalis* only compared to those dually treated with bacteria and CBD in WT animals only, all *p* < 0.05. The IgM signal was generally lower in CB2-deficient animals compared to CB2 replete mice. */**p ≤ 0.05/0.01, with all groups compared by ANOVA.

*P. gingivalis* is a key periodontal pathogen in humans. However, the mouse is not a natural host of this bacterium. Therefore, chronic colonization can be difficult. Further, C57Bl6 is not an ideal mice strain for bone loss experiments. Therefore, our model is one of repeated transient *P. gingivalis* infection insufficient to induce the loss of alveolar bone surrounding the teeth, but appropriate for the induction of a robust immune response in the requisite CB2^−/−^ genetic background (see Figure 7). Interestingly, CBD treatment was associated with suppressed total IgM production in wild type but not CB2^−/−^ mice (Figures 7A,B). On the other hand, total IgG and *P. gingivalis*-cognizant IgM or IgG were not influenced by CBD administration (*data not shown*). The *P. gingivalis*-elicited mRNA signal for the pro-inflammatory cytokine, IL-1β (Figures 7C,D), the macrophage marker, CD14 (Figures 7E,F), and the pan-leukocyte marker, CD45 (Figures 7G,H), but not the CXCL2 murine analog Mip-2 or MMP-8 (*data not shown*), were each suppressed by CBD in periodontal tissues of wild type but not CB2 knockout mice. Thus, in a transient *P. gingivalis* infection model, CBD suppressed components of both the humoral and innate immune response *in vivo*. If such phenomena occur chronically in human marijuana users, natural *P. gingivalis* hosts, it can be imagined that cannabinoid exposure could set up conditions that predispose to periodontal disease, a condition with a normally prolonged onset.

## Discussion

In order to prevent bacterial dissemination on the one hand and collateral tissue damage on the other, the inflammatory response to dental plaque, triggered upon the recognition of microbe-associated molecular patterns by pattern recognition receptors on innate immune cells, must be tightly regulated. Cigarette smoke is known to dysregulate such host-pathogen interactions, for example, by suppressing pro-inflammatory cytokine production and angiogenesis, helping to explain the dysbiotic microbiota and increased incidence and severity of chronic periodontitis that is consistently noted in tobacco smokers relative to non-smokers (52, 53). Increasing evidence suggests that inhalation of cannabis smoke is also negatively associated with periodontal health (5, 10, 12, 54). The mechanisms of predisposition are unknown, but marijuana has long been attributed anti-inflammatory properties (49, 50). To the best of our knowledge, we are the first to report on the influences of multiple marijuana-derived cannabinoids on the innate response to specific oral bacteria in monocytes and epithelial cells *in vitro* and in mice *in vivo*.

The mean cannabis concentrations of CBD, CBN, and THC have been reported as 0.3, 0.3, and 3.1%, respectively (55), while Ki affinities for the primary innate cannabinoid receptor, CB2, are 4000, 150, and 25 nM, respectively (48). The median (range) concentrations of CBD, CBN and THC in the saliva of marijuana smokers have been reported as 21 (4.5–255) ng/ml, 37 (16–476) ng/ml, and 517 (189–6,508) ng/ml, respectively, while concentrations in oral fluid can increase 10–20-fold in the immediate aftermath of smoking (56). Chen et al. have reported that the nicotine metabolite, cotinine, may be found in the gingival crevicular fluid of smokers at higher concentrations (× 4) than in saliva (57), although contrasting data also exists (58). The potential for sequestration or concentration of phytocannabinoids in the gingival crevice has yet to be ascertained. With this in mind, we employed CBD, CBN, and THC at doses deemed likely to occur *in situ* (0.1–10 μg/ml), primarily, 1.0 μg/ml.

While it is clear that inhalation of cigarette smoke exerts a profound influence on the composition of dental plaque (52, 59, 60), the potential effects of marijuana on the oral microbiota remain unknown. Herein, we show that high doses of CBD, CBN, and THC each inhibit the growth of two oral pathobionts, *P. gingivalis* and *F. alocis*, but not *T. denticola*. These data suggest that cannabis use has the potential to induce shifts in the composition or balance of oral microbes. Should phytocannabinoid intake similarly favor the survival of selected bacterial species in the oral cavity in humans, a phenomenon yet to be established, then there is potential for marijuana to contribute to the microbial dysbiosis that is a hallmark of periodontitis.

At cannabinoid doses at the high end of those likely to occur *in vivo*, the viability of two key human innate cell types, epithelial cells and monocytes, was compromised. Such innate cells are essential for the detection of bacterial infection and the subsequent initiation and progression of an inflammatory response that is capable of protecting the periodontium against, or clearing, the microbial insult. While limited relevant toxicological data are available, high doses of CB receptor agonists have been recently suggested also to be cytotoxic to human periodontal ligament fibroblasts (61). Again, should these phenomena occur in humans *in situ*, cannabinoids could also predispose to periodontal diseases through compromised barrier function and innate capacity.

CBD, CBN, and THC each efficiently suppressed IL-6, IL-8, IL-12 p40, and TNF release from bacteria-stimulated innate cells while enhancing production of the anti-inflammatory cytokine, IL-10. In tobacco smokers, reduced overt clinical inflammation of the gingiva associated with pro-inflammatory cytokine suppression occurs concomitantly with increased periodontitis susceptibility, a phenomenon referred to as the clinical conundrum (62, 63). Inflammation is a composite of hundreds of events. Thus, while tobacco smoking dampens the cytokine arm of the innate response to pathogens, other aspects of inflammation are not affected or are upregulated, such as the burden of multiple inflammatory mediators and biomarkers, e.g., CRP, sICAM-1, MMP-8, myeloperoxidase, and neutrophil elastase, as we have recently detailed (52). It is tempting, but too early, to speculate that similar events may occur in cannabis smokers. Certainly, the influence of cannabis on vascular-related indices in the oral cavity, if available, are difficult to extract from the published literature. Nevertheless, vasoconstriction may be a general cannabinoid-related physiological endpoint, as recently reviewed by Richter et al. (64). A reduced vasoreactivity to dental plaque alongside a reduced pro-/enhanced anti-inflammatory cytokine response *in vivo* would be expected to promote pathogen persistence in the subgingival environment, potentially promoting a highly chronic, low grade inflammation.

Our pro-inflammatory cytokine suppression data are in keeping with, but extend, prior reports that, for example, a single cannabinoid (CBD) bolus reduced TNF and IL-6 concentrations, as well as myeloperoxidase activity, in a LPS-induced acute lung injury mouse model (44). CBD has also been shown to suppress IL-1β production in a mouse viral-induced demyelinating disease model, while reducing vascular activation as determined by monitoring endothelial VCAM-1 expression (43). As for the periodontium itself, Nakajima et al. have reported that LPS-triggered NF-κB activation, the key event in the induction of pro-inflammatory cytokine expression, is blocked in gingival fibroblasts by the endocannabinoid, anandamide (65), while the synthetic anandamide analog, methanandamide, has been shown to suppress local TNF production and reduce alveolar bone loss and in an LPS-injection model of periodontitis (66). Also of relevance is the recent report by Abidi et al. suggesting that the CB2 agonist, HU-308, suppresses *P. gingivalis* LPS-induced IL-6 release from primary human periodontal ligament fibroblasts (61).

Perhaps the most pertinent mechanistic data related to the immunosuppressant activities of cannabinoids has been reported by Juknat et al. (67) who reported that CBD and THC each activate immunoregulatory genes within the MAPK (Dusp1, Dusp8, Dusp2) and JNK/STAT (Socs3, Cish, Stat1) families in myeloid lineage cells, microglia. Unlike THC, CBD was shown to induce Trib3 and Dusp1, negative regulators of NF-κB and AP-1. Also in microglia, Kozela et al. (68) have shown that THC and CBD suppress LPS-induced IL-1β and IL-6 but only CBD influenced NFκB activity. Thus, there is evidence for differential mechanisms of immunoregulation between cannabinoid subtypes and cell types.

The PI3K-GSK3β signaling axis represents a critical endogenous anti-inflammatory circuit that functions to limit the innate response to pathogens, thus protecting from inflammation-driven tissue damage and septic shock. The best characterized endogenous trigger of PI3K-GSK3β signaling is acetylcholine. However, engagement of α7 nicotinic acetylcholine receptors by tobacco-derived alkaloids (nicotine or cotinine) potently augments the GSK3β-mediated shut down of bacterial-induced inflammation. In short, α7nAChR agonists activate PI3K; leading to phospho-inactivation of GSK3β at Ser9 (via Akt and PIP_3_) resulting in increased activity of CREB, displacement of NF-κB p65 from the co-activator of transcription, CBP, and lowered transcriptional activity of NF-κB p65-driven pro-inflammatory genes, as we have recently reviewed (36). Additionally, CBD has been reported to be a PI3K activator and GSK3β downregulator in the context of neuronal dysfunction (38). Therefore, we examined if engagement of innate cell cannabinoid receptors similarly activated the PI3K-GSK3β signaling axis.

Pharmaceutical inhibition of cannabinoid receptor 2 (CB2) with JTE907, combined with CB1 and CB2 gene silencing, indicated that phytocannabinoid-mediated immune suppression acts via CB2 not CB1. Further, diminished pro-inflammatory cytokine production was PI3K- but not GSK3β-dependent, again as determined by both pharmaceutical inhibition (LY294002; SB216763; and LiCl, respectively) and gene silencing (PI3K p110δ/p85α; GSK3β). These data suggest that a common CB2/PI3K axis of immune suppression is triggered by CBD, CBN, and THC. However, the anti-inflammatory signal does not appear to perpetuate through the canonical, GSK3β-dependent cholinergic anti-inflammatory pathway.

CBD did not influence bone loss (CEJ-ABC; volume or density) in WT or CB2^−/−^ mice (*data not shown*). However, *P. gingivalis* is not a natural murine pathogen, while C57Bl6 mice represent a less periodontitis-susceptible strain with the requisite CB2 ablation. Our model, therefore, is one of a repetitive, transient *P. gingivalis*-infection sufficient to induce an inflammation in the oral environment. Importantly, then, CBD appears to suppress the immune response to *P. gingivalis* in mice in a CB2-dependent manner. Suppressed *P. gingivalis*-induction of CD45, CD14, and IL-1β are suggestive of a reduced inflammatory environment in the periodontium of CBD-treated mice. If applicable to the human oral environment, this may compromise immune clearance of oral pathogens, which would likely increase susceptibility to destructive periodontal diseases, particularly over the long term. As noted earlier, periodontitis is a highly chronic disease not usually manifest until middle age and later.

Interestingly, B cells have been reported to express the highest level of CB2 among the leukocyte subsets (69), while we show that CBD suppresses total IgM, but not IgG, in CB2-replete but not knockout mice. Thus, the primary humoral response is compromised by CBD delivery in mice. CBD has previously been shown to suppress the ovalbumin-elicited OVA-cognizant IgM response in Balb/C mice (70). Springs et al. have reported that THC effectively suppressed the T cell-dependent, anti-sheep erythrocyte IgM antibody-forming cell response in wild-type but not in ${\text{CB}}_{1}^{-/-}$/${\text{CB}}_{2}^{-/-}$ double knockout mice (71). There is also precedent literature suggesting that marijuana use may be associated with immunoglobulin class switching, with IgA increased in pre- and neonatally exposed children (72). Also, a pan-cannabinoid receptor agonist, but not a CB1-specific activator, has been reported to induce an IgM to IgE switch in murine splenic B cells (73). On the other hand, Simkins et al. have reported an elevated basal or LPS-induced IgM titer in CB1/CB2 double knockout mice (74). While the mechanisms by which cannabionoids may influence antibody production remain elusive, it has been established that there is a reduction in mature B cell migration to, and retention by, the splenic marginal zone in mice lacking CB2 (69). Others have suggested that THC reduces the number of IgM-producing cells that can be differentiated from human peripheral B cells (75). A compromised primary antibody response, in humans, could promote periodontal diseases by facilitating colonization during dysbiotic shifts or the assimilation of new pathogens into dental plaque.

In summary, this study provides unique information on three phytocannabinoid subtypes, their antimicrobial properties and their cytotoxic and immunosuppressive influences of on oral bacteria-exposed human monocytes and epithelial cells. More specifically, a common CB2/PI3K axis of immune suppression appears to be triggered by CBD, CBN, and THC. The anti-inflammatory signal does not appear to perpetuate through the conventional GSK3β-dependent cholinergic anti-inflammatory pathway. If these phenomena occur in humans *in vivo*, then phytocannabinoids could enhance chronic periodontitis, at least in part, by promoting microbial dysbiosis through direct toxic effects on specific oral bacteria or by aiding initial colonization through compromised IgM production; by compromising innate cell vitality; and/or through a suppressed innate response to periodontal pathogens.

## Ethics Statement

All experimental procedures were performed in accordance with the Guidelines of the Institutional Review Board and Institutional Animal Care and Use Committee of University of Louisville and as specified in IRB #12.0346 and IACUC 15434, respectively.

## Author Contributions

DS designed the experiments, contributed to the analysis of the data, and drafted the article. ZG, RN, SS, and GL performed the experiments and contributed to data analysis. RL and HW assisted with data interpretation and contributed critical revisions to the manuscript.

### Conflict of Interest

The authors declare that the research was conducted in the absence of any commercial or financial relationships that could be construed as a potential conflict of interest.
